# Occupational stress, moral dilemmas, and decision-making confidence among Chinese police officers: the mediating role of emotional exhaustion

**DOI:** 10.3389/fpsyg.2026.1971012

**Published:** 2026-09-30

**Authors:** Yi Li

**Affiliations:** Mental Health Education Center, Henan Police College, Zhengzhou, China

**Keywords:** Chinese police officers, decision confidence, emotional exhaustion, mediation, moral dilemma, occupational stress, police decision-making

## Abstract

Police officers routinely make consequential decisions in emotionally demanding and morally ambiguous situations, yet how chronic occupational stress and moral strain relate to the confidence with which officers reach those decisions remains poorly specified. This cross-sectional study examined whether the associations of occupational stress and moral dilemmas with decision-making confidence among Chinese police officers are statistically accounted for by emotional exhaustion within a hypothesized mediation model. A convenience sample of 412 active-duty officers from three municipal police bureaus in Henan Province, China, completed validated Chinese versions of the Operational Police Stress Questionnaire, a six-item moral dilemma scale, the emotional exhaustion subscale of the Maslach Burnout Inventory-General Survey, the 12-item General Health Questionnaire, and a five-item decision-making confidence scale. Structural equation modeling with 5,000 bias-corrected bootstrap resamples showed that both occupational stress (β = 0.41, *p* < 0.001) and moral dilemmas (β = 0.27, *p* < 0.001) were positively associated with emotional exhaustion, which was in turn negatively associated with decision-making confidence (β = −0.38, *p* < 0.001). The indirect effects of occupational stress (indirect effect = −0.16, 95% CI [−0.21, −0.11]) and moral dilemmas (indirect effect = −0.10, 95% CI [−0.15, −0.06]) through emotional exhaustion were both statistically significant, whereas the direct effects of the two antecedents were non-significant. The model explained 38% of the variance in emotional exhaustion and 15% of the variance in decision-making confidence. These findings are consistent with resource-based interpretations grounded in conservation of resources theory, and they identify emotional exhaustion as a potentially modifiable correlate of officers’ self-evaluated decisional capacity; whether reducing burnout also benefits decision-making itself remains to be established with longitudinal and experimental designs.

## 1 Introduction

Police decision-making unfolds in emotionally charged, uncertain, and time-pressured environments in which officers must interpret ambiguous cues, regulate affect, assess risk, and act in ways that are lawful, proportionate, and operationally effective (Fridman et al., 2019). Such decisions are not shaped by cognition alone. Emotional states, chronic occupational stress, fatigue, burnout, trauma exposure, and moral strain can all influence attention, judgment, impulse control, and post-incident recovery (Nieuwenhuys et al., 2012a; Wu et al., 2021). Policing is widely recognized as one of the most stressful occupations, and officers in China are no exception: with fewer than one officer per 10,000 population in many jurisdictions and heavy workloads compounded by rigid bureaucracy, shift work, and exposure to critical incidents, Chinese officers report substantial levels of stress and psychological distress (Wu and Wen, 2020; Huang et al., 2021; Zhou et al., 2024). A large provincial survey of more than 40,000 officers documented clinically significant mental health risks among a substantial minority of Chinese police personnel, confirming that psychological strain is a systemic feature of contemporary Chinese policing rather than an isolated phenomenon (Teng, 2025).

Within this high-demand occupational context, the quality of police decisions carries consequences that extend far beyond individual performance to public trust, procedural justice, use-of-force outcomes, and officers’ long-term mental health (Liu, 2023). Experimental work on police shooting decisions has demonstrated that anxiety and threat reliably bias officers toward premature or erroneous responses, shifting them from objective visual information toward threat-related inferences and expectations (Nieuwenhuys et al., 2012a, 2012b, 2015). These laboratory findings align with ecological accounts of constructed emotion, which hold that all mental events, including perception and judgment, are embodied and predictive, and that autonomic arousal directly shapes behavior under stressful circumstances (Fridman et al., 2019). Yet most of this evidence has been generated in Western samples and in controlled settings. Far less is known about how chronic, everyday occupational strain, rather than acute laboratory threat, is associated with officers’ confidence in their real-world decisions, particularly in non-Western police systems such as China.

Two occupational characteristics of Chinese policing make it an especially informative setting for examining this question. First, Chinese officers face persistent organizational stressors, including administrative overload, hierarchical management, and limited resources, alongside operational stressors such as shift work, public order maintenance, and exposure to traumatic events (Wu and Wen, 2020; Zhou et al., 2024). Large-scale surveys confirm that these stressors translate into measurable psychological costs: in a provincial sample of more than 40,000 officers, a substantial proportion screened positive for mental health problems on the GHQ-12, and stress-related morbidity clustered geographically with environmental and workload indicators (Teng, 2025). Meta-analytic evidence from 39 Chinese police studies further shows a robust positive correlation between work pressure and occupational burnout (r = 0.41), with negative coping styles operating as a partial mechanism (Zhou et al., 2024). Chinese officers are also chronically understaffed relative to their workloads, which intensifies shift demands, overtime, and exposure to critical incidents (Wu and Wen, 2020; Yuan et al., 2020).

Second, officers frequently encounter morally demanding situations, from discretionary use-of-force judgments to enforcement decisions involving vulnerable populations, in which conflicting duties and values must be balanced under scrutiny. Research on moral judgment distinguishes personal from impersonal moral dilemmas, with personally involving dilemmas eliciting stronger emotional engagement (Greene et al., 2001); the enforcement conflicts officers report are frequently of this personally involving kind, because they carry direct consequences for identifiable citizens. Qualitative research with Chinese special tactical police has documented the moral ambivalence that routine enforcement can generate and the psychological effort officers invest in reconciling conflicting moral perspectives, either by deferring to the legitimacy of higher-level decision-making or by disengaging from personal moral deliberation, a pattern consistent with the moral disengagement processes described by Bandura (1999) and Liu (2023). Among law enforcement officers more generally, moral injury, the distress arising from perpetrating, failing to prevent, or witnessing acts that transgress deeply held moral beliefs, has emerged as a distinct predictor of trauma-related symptomatology over and above conventional stressors and compassion fatigue (Papazoglou et al., 2020). These moral demands may therefore constitute an independent source of psychological strain that is not captured by measures of workload or organizational stress, and systematic reviews of police mental health have identified exposure to morally stressful events as a key risk factor for adverse psychological outcomes (Sherwood et al., 2019). The stress experienced by Chinese officers is compounded by the visibility of contemporary enforcement: public scrutiny, media coverage, and expectations of procedural fairness heighten the perceived stakes of every discretionary decision, and officers who fear that their judgment will be questioned report additional psychological burden (Huang et al., 2021; Wu et al., 2021).

Although occupational stress and moral dilemmas are modeled here as parallel antecedents, they differ in their sources and temporal dynamics. Occupational stress aggregates sustained operational and organizational demands, such as workload, shift schedules, and bureaucratic constraints, whereas moral dilemmas arise from discrete events in which duties, values, or loyalties conflict. Their cognitive-affective routes also differ: operational demands are appraised primarily in terms of workload and resource strain, whereas moral conflicts engage personal values and standards. The two classes of demands can nevertheless converge on the same depleted state: within the challenge-hindrance perspective on occupational stress (Cavanaugh et al., 2000), hindrance demands of different origins accumulate into strain when they jointly exceed available resources, a logic shared by COR theory and the JD-R model. Modeling the two antecedents in parallel is therefore a theory-based choice rather than a claim of equivalence, and their distinctiveness is evaluated empirically through the discriminant-validity analyses reported in Section 3.4.

The consequences of strain for police judgment are well documented at the acute level. In simulated shooting tasks, experimentally induced anxiety increases officers’ bias toward firing, reduces accuracy, and shifts responding from objective visual information toward threat-related expectations (Nieuwenhuys et al., 2012a, 2012b, 2015). These effects persist despite practice, suggesting that threat-induced decision errors are robust and difficult to eliminate through training alone (Nieuwenhuys et al., 2015). Ecological accounts of constructed emotion explain such findings by arguing that all judgment is embodied and predictive: interoceptive signals from the body continuously shape perception and action, and under arousal, perception becomes biased toward threat (Fridman et al., 2019). What remains less clear is whether chronic occupational strain, the accumulated residue of months and years of operational and moral demands, exerts a similarly corrosive influence on officers’ confidence in their everyday judgments. Decision-making confidence, the self-assuredness with which officers reach and stand behind their decisions, is a psychologically meaningful outcome in its own right: it sustains decisive action under time pressure, supports communication with citizens and colleagues, and protects officers from the ruminative self-doubt that characterizes post-incident distress (Bandura, 1997; Nieuwenhuys et al., 2015). In the judgment and decision-making literature, confidence is examined alongside calibration and overconfidence, and self-reported confidence is recognized as distinct from decision accuracy (Moore and Healy, 2008); the construct studied here concerns officers’ global self-evaluation of their decisional capacity rather than calibrated confidence in specific judgments.

The mechanisms linking such strain to decision-making remain underspecified. Conservation of resources (COR) theory provides a parsimonious account: individuals strive to protect, retain, and accumulate resources, and when work demands chronically deplete those resources, strain and burnout ensue (Hobfoll, 1989, 2001). Within the job demands-resources (JD-R) framework, emotional exhaustion, the core component of burnout, represents the depletion of energetic resources that results when demands exceed resources (Demerouti et al., 2001; Maslach et al., 2001; Bakker and Demerouti, 2017). Longitudinal evidence from Chinese occupational samples, including police officers, shows that increases in job demands are followed by increases in burnout, whereas job resources buffer this process (Hu et al., 2017). Critically, a depleted energetic state should not stop at burnout itself: resource-depleted individuals have fewer attentional and regulatory resources available for effortful judgment, and self-efficacy beliefs, the confidence in one’s capability to execute required actions, are themselves resource-sensitive and predictive of performance persistence (Bandura, 1997). Decision-making confidence, defined here as officers’ self-assuredness in their capacity to make sound, lawful, and proportionate judgments in operational and everyday contexts, should therefore be vulnerable to the resource depletion indexed by emotional exhaustion. Attentional control theory similarly predicts that anxiety consumes attentional resources and degrades processing efficiency in high-stakes tasks (Eysenck et al., 2007), and police-specific experimental evidence indicates that anxious states increase response bias and reduce decision accuracy (Nieuwenhuys et al., 2012a, 2012b). Emotional exhaustion, as the chronic residue of accumulated stress and moral strain, Within the JD-R framework, moral dilemmas can accordingly be treated as a distinct class of job demand that is appraised through value conflict rather than through workload, complementing the operational and organizational demands that dominate conventional stress measures (Demerouti et al., 2001; Bakker and Demerouti, 2017). Emotional exhaustion, as the chronic residue of accumulated stress and moral strain, plausibly operates on decision confidence through the same resource-based logic.

Despite the theoretical coherence of this account, direct empirical tests among police officers are scarce. Existing Chinese police research has separately documented the prevalence of occupational stress and burnout (Wu and Wen, 2020; Zhou et al., 2024), the association between stress and psychological distress (Huang et al., 2021; Yuan et al., 2020), and the moral dimensions of enforcement work (Liu, 2023), but no study to our knowledge has modeled emotional exhaustion as the mediating variable in the associations of both occupational stress and moral dilemmas with decision-making confidence. In addition, the moral dilemma construct has rarely been quantified in survey-based police research, and its incremental contribution to burnout beyond conventional occupational stress has not been established. Testing this integrative model has both theoretical value, clarifying whether moral strain operates through the same resource-based logic as workload stress, and practical value, identifying emotional exhaustion as a common, modifiable target for protecting decision confidence in policing.

The Chinese context also offers a distinctive opportunity to examine the moral dimension of police work. Chinese officers operate within a legal and organizational framework that emphasizes both strict law enforcement and service to the public, and contemporary reforms toward standardized, transparent, and accountable enforcement have heightened the moral and professional stakes of individual decisions (Liu, 2023). Front-line officers in particular must reconcile formal legal requirements, performance targets, community expectations, and their own ethical standards in situations that are frequently ambiguous, and the psychological cost of such reconciliation has been documented in qualitative accounts of moral ambivalence among Chinese tactical and public order officers (Liu, 2023). Survey-based quantification of moral dilemmas, as attempted in the present study, is a necessary complement to these qualitative insights, because it allows the incremental contribution of the moral dimension to be tested against well-established occupational stressors within a single statistical model.

Conceptually, decision-making confidence is best understood not as a trait-like disposition but as a state that is sensitive to available cognitive and emotional resources. Self-efficacy theory holds that confidence beliefs are built from mastery experiences, vicarious learning, social persuasion, and physiological states, and that depleted physiological and emotional states weaken efficacy beliefs (Bandura, 1997). Attentional control theory adds that anxiety consumes the executive resources required for efficient processing, degrading performance particularly under high cognitive load (Eysenck et al., 2007). Police-specific evidence demonstrates that acute anxiety biases shooting decisions and that such biases are persistent (Nieuwenhuys et al., 2012a, 2012b, 2015). Extending this logic, emotional exhaustion, as the chronic depletion of energetic resources, should produce a sustained rather than transient erosion of the confidence officers place in their judgments. This extension is the central theoretical claim of the present study.

Accordingly, the present study examined a mediation model in which occupational stress and moral dilemmas are associated with lower decision-making confidence through elevated emotional exhaustion among Chinese police officers (Figure 1). Three hypotheses were advanced. H1: Occupational stress positively predicts emotional exhaustion. H2: Moral dilemmas positively predict emotional exhaustion over and above occupational stress. H3: The negative associations of both occupational stress (H3a) and moral dilemmas (H3b) with decision-making confidence operate through emotional exhaustion, such that the direct effects of the two antecedents on decision confidence are non-significant after accounting for the mediator. Testing these hypotheses in a Chinese officer sample addresses a gap identified across the occupational health and police decision-making literatures, and it aligns directly with the priorities of contemporary police psychology research on the emotional and motivational processes underlying judgment under pressure (Fridman et al., 2019).

**Figure 1:**
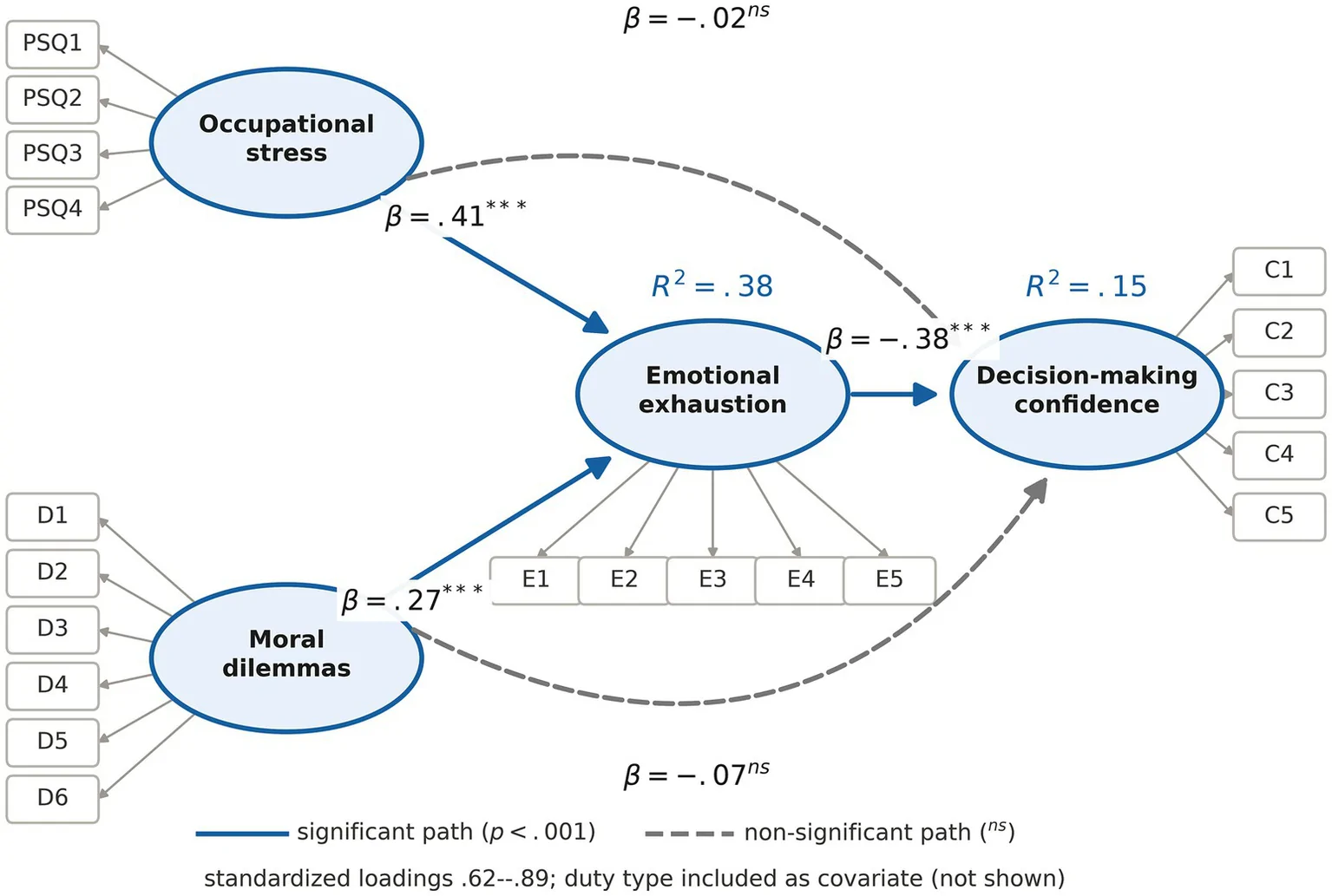
Mediation model of occupational stress and moral dilemmas on decision-making confidence through emotional exhaustion. Standardized coefficients are reported; duty type was included as a covariate. Solid lines represent significant paths (***p < 0.001); dashed lines represent non-significant direct paths. R^2^ = 0.38 for emotional exhaustion; R^2^ = 0.15 for decision-making confidence.

## 2 Materials and methods

### 2.1 Research design and participants

A cross-sectional questionnaire survey was conducted between March and June 2026 among active-duty police officers in Henan Province, China. Participants were recruited through convenience cluster sampling at three municipal public security bureaus (Zhengzhou, Luoyang, and Nanyang) in collaboration with the psychological health service units of the respective bureaus. Inclusion criteria were current employment as a sworn police officer, at least one year of service, and active engagement in operational duties. Officers were invited to participate through their units’ internal psychological health education channels; participation was voluntary and anonymous, and no incentive was offered. A total of 471 questionnaires were distributed and 446 were returned (response rate 94.7%). After excluding 34 cases for incomplete responses (more than 10% missing items), straight-lining across all substantive items, or failing the embedded instructed-response check item, the final sample comprised 412 officers (effective rate 87.5%). Of these, 152 officers were from the Zhengzhou bureau, 134 from the Luoyang bureau, and 126 from the Nanyang bureau.

The sample included 331 males (80.3%) and 81 females (19.7%), aged 22 to 51 years (M = 34.6, SD = 7.8); mean years of service was 11.4 (SD = 8.2). Regarding rank, 128 (31.1%) were constables, 176 (42.7%) sergeants, and 108 (26.2%) inspectors or above. Front-line duties (patrol, traffic, and public order) accounted for 268 officers (65.0%), and non-front-line administrative or support duties for 144 (35.0%). Administrative and support personnel in the three bureaus periodically deploy for operational duties, for example during large-scale security operations; officers were therefore classified by primary duty type.

### 2.2 Instruments

All instruments were administered in Chinese. The English source instruments were translated and adapted following Brislin's (1970) back-translation procedure by two bilingual researchers with doctoral training in psychology, and discrepancies were resolved through discussion. Unless otherwise noted, item responses were averaged to form scale scores, with higher scores indicating higher levels of the measured construct. A pilot study with 58 officers drawn from the same population but excluded from the main sample supported the reliability and clarity of all instruments.

Occupational stress was measured with the Operational Police Stress Questionnaire (PSQ-Op; McCreary and Thompson, 2006), a 20-item instrument assessing stressors inherent to police work, such as shift work, exposure to traumatic scenes, occupational danger, and work-family interference. Each item was rated on a seven-point scale from 1 (not at all stressful) to 7 (very stressful). The PSQ has demonstrated strong reliability and validity across international police samples (McCreary and Thompson, 2006). In the present study, Cronbach’s α = 0.93.

Moral dilemmas in policing were assessed with a six-item scale developed for this study on the basis of the moral injury literature (Papazoglou et al., 2020) and qualitative evidence from Chinese police settings (Liu, 2023). The construct was defined as the frequency of encountering situations in which duties, values, or loyalties conflict; it is conceptually distinct from moral distress and moral injury, which refer to the psychological aftermath of such events rather than to the exposure itself (Papazoglou et al., 2020). Items captured the frequency with which officers encountered situations in which competing duties, values, or loyalties conflicted and in which acting in accordance with one duty necessarily violated another, for example, enforcing a policy that the officer judged harmful to a vulnerable citizen, balancing aggressive enforcement targets against the interests of minor offenders, and witnessing or participating in actions that conflicted with personal moral standards (e.g., “In the past six months, how often did you face situations in which following orders conflicted with what you personally believed was right?”). Each item was rated on a five-point scale from 1 (never) to 5 (very often). Confirmatory factor analysis supported a unidimensional structure (CFI = 0.97, RMSEA = 0.06); Cronbach’s α = 0.84. The complete item wording is provided in the Supplementary Material.

Emotional exhaustion was measured with the five-item emotional exhaustion subscale of the Chinese version of the Maslach Burnout Inventory-General Survey (MBI-GS; Maslach and Jackson, 1981; Li and Shi, 2003). Items were rated on a seven-point frequency scale from 0 (never) to 6 (every day). The Chinese MBI-GS was revised and validated for use with Chinese occupational samples (Li and Shi, 2003). In the present study, Cronbach’s α = 0.90.

Psychological distress was assessed with the 12-item General Health Questionnaire (GHQ-12; Goldberg and Williams, 1988), which served as a concurrent validity indicator of the strain process. The Chinese version of the GHQ-12 has demonstrated satisfactory reliability in general and occupational Chinese populations (Wojujutari et al., 2024) and has been used in large-scale Chinese police surveys (Teng, 2025). In the present study, Cronbach’s α = 0.88.

Decision-making confidence was measured with a five-item scale developed for this study, following theoretical accounts of confidence beliefs in high-stakes judgment (Bandura, 1997) and informed by experimental research on decision-making under pressure (Nieuwenhuys et al., 2015). The construct was conceptualized as a self-reported evaluation of one’s decisional capacity, distinct from decision accuracy, confidence calibration, decisiveness, and general self-efficacy (Moore and Healy, 2008). Items assessed officers’ self-assuredness in their capacity to reach sound judgments across routine and critical situations, to remain decisive under time pressure, to apply law and policy proportionately, and to trust their own judgment after the fact (e.g., “Even in uncertain situations, I am confident in my ability to make the right decision”; “After making a decision, I rarely doubt whether it was correct”). Items were rated on a five-point scale from 1 (strongly disagree) to 5 (strongly agree). Confirmatory factor analysis supported a unidimensional structure (CFI = 0.98, RMSEA = 0.05); Cronbach’s α = 0.86. The complete item wording is provided in the Supplementary Material.

Demographic information included gender, age, years of service, rank, and duty type (front-line vs. non-front-line).

A Monte Carlo simulation for the hypothesized mediation model (5,000 replications using the expected parameter values of β = 0.41, β = 0.27, and β = −0.38) indicated that a sample of 412 provided power above 0.95 for detecting the smallest expected indirect effect at α = 0.05. The final sample therefore exceeded the minimum size recommended for stable structural equation modeling with four latent factors (Kline, 2023), and the ratio of cases to free parameters was approximately 9:1, which is adequate for robust maximum likelihood estimation.

### 2.3 Procedure

Questionnaires were administered anonymously through the Wenjuanxing online survey platform. Each participating bureau distributed a unique survey link through its internal psychological health service channel during routine training sessions. Completion took approximately 20 min. An instructed-response item (“Please select “often” for this item”) was embedded in the questionnaire as an attention check, and a final debriefing page informed participants of the study purpose and contact details of the first author for questions or psychological support referrals.

### 2.4 Ethics statement

The studies involving human participants were reviewed and approved by the Academic Ethics Committee of Henan Police College (approval number HPEC-2026-018). The study was conducted in accordance with the Declaration of Helsinki. All participants provided written informed consent after being informed of the purpose, voluntariness, anonymity, and data use of the survey. Data were collected anonymously, stored on encrypted devices, and reported only in aggregate form. No individual-level data were shared with the participating bureaus.

### 2.5 Data analysis

Analyses were conducted with SPSS 26.0 and Mplus 8.3. Preliminary screening examined missing data, univariate outliers, and common method variance via Harman’s single-factor test (Podsakoff et al., 2003). Descriptive statistics, Cronbach’s alphas, and Pearson correlations were computed for all study variables. The hypothesized mediation model was then estimated with structural equation modeling (SEM) using robust maximum likelihood estimation. Missing data were handled using full information maximum likelihood estimation.

The measurement model comprised four latent factors (occupational stress, moral dilemmas, emotional exhaustion, decision-making confidence) with item parcels serving as indicators; parceling was applied to the 20-item PSQ-Op (four parcels of five items) to keep the indicator-to-sample ratio tractable, whereas the shorter scales were modeled with their individual items. Model fit was evaluated against the criteria of CFI and TLI ≥ 0.90 (preferably 0.95), RMSEA ≤ 0.08, and SRMR ≤ 0.08 (Hu and Bentler, 1999). Convergent and discriminant validity were assessed with composite reliability (CR), average variance extracted (AVE), and the Fornell-Larcker criterion (Fornell and Larcker, 1981).

Indirect effects were tested with 5,000 bias-corrected bootstrap resamples; an indirect effect was considered significant when its 95% confidence interval excluded zero (Preacher and Hayes, 2008; Hayes, 2022). Gender, age, years of service, and duty type were examined as covariates and retained if significantly associated with the mediator or the outcome.

## 3 Results

### 3.1 Demographic characteristics

Table 1 presents the demographic distribution of the final sample (*N* = 412). The sample was predominantly male (80.3%), with an average age of 34.6 years (SD = 7.8) and an average length of service of 11.4 years (SD = 8.2). Sergeants accounted for the largest proportion of rank (42.7%), and front-line operational duties made up 65.0% of the sample.

**Table 1:** Demographic characteristics of participants (*N* = 412).

Variable	Category	Frequency	Percentage (%)
Gender	Male	331	80.3
	Female	81	19.7
Age (years)	20–29	112	27.2
	30–39	187	45.4
	40–49	105	25.5
	≥50	8	1.9
Years of service	1–5	101	24.5
	6–15	174	42.2
	16–25	118	28.6
	≥26	19	4.6
Rank	Constable	128	31.1
	Sergeant	176	42.7
	Inspector or above	108	26.2
Duty type	Front-line (patrol/traffic/public order)	268	65.0
	Non-front-line (administrative/support)	144	35.0

### 3.2 Common method bias test

Harman’s single-factor test was conducted as a descriptive check of common method variance. An unrotated exploratory factor analysis on all substantive items extracted six factors with eigenvalues greater than 1, with a cumulative variance explanation rate of 62.3%, and the first factor accounted for 28.4% of the total variance (Podsakoff et al., 2003). This test is reported descriptively; it does not rule out common method variance, which is treated as an unresolved limitation (see Section 4.1).

### 3.3 Descriptive statistics and correlation analysis

Table 2 presents the means, standard deviations, internal consistency coefficients, and Pearson correlations for all study variables. Occupational stress (M = 3.84, SD = 0.96) and emotional exhaustion (M = 2.13, SD = 1.18) levels were consistent with those reported in prior Chinese police samples. The prevalence of probable psychological distress (GHQ-12 score ≥ 4) was 31.8%, aligning with provincial epidemiological data.

**Table 2:** Descriptive statistics, internal consistency, and intercorrelations among study variables (*N* = 412).

Variable	M	SD	α	1	2	3	4	5
1. Occupational stress	3.84	0.96	0.93	—				
2. Moral dilemmas	2.71	0.82	0.84	0.38**	—			
3. Emotional exhaustion	2.13	1.18	0.90	0.49**	0.41**	—		
4. Decision-making confidence	3.62	0.71	0.86	−0.26**	−0.24**	−0.38**	—	
5. Psychological distress	3.05	2.58	0.88	0.35**	0.28**	0.44**	−0.25**	—

M = mean; SD = standard deviation; α = Cronbach’s alpha coefficient. **p < 0.01.

All scales demonstrated good internal consistency, with Cronbach’s α values ranging from 0.84 to 0.93. Occupational stress and moral dilemmas were both positively correlated with emotional exhaustion (*p* < 0.001), and emotional exhaustion was negatively correlated with decision-making confidence (*p* < 0.001). Psychological distress (GHQ-12) showed the expected pattern of correlations with core study variables, providing concurrent validity support.

Among the demographic variables, only duty type was significantly associated with the study variables: front-line officers reported higher occupational stress (t = 3.12, *p* = 0.002) and emotional exhaustion (t = 2.47, *p* = 0.014) than non-front-line officers, and duty type was therefore retained as a covariate in subsequent structural equation analyses.

### 3.4 Measurement model and psychometric properties

Confirmatory factor analysis was conducted to evaluate the hypothesized four-factor measurement model (occupational stress, moral dilemmas, emotional exhaustion, decision-making confidence). As shown in Table 3, the four-factor model demonstrated good fit to the data: χ^2^(164) = 378.8, χ^2^/df = 2.31, CFI = 0.95, TLI = 0.94, RMSEA = 0.056 (90% CI [0.048, 0.064]), SRMR = 0.045.

**Table 3:** Fit indices of the measurement model and alternative models.

Model	χ^2^	df	χ^2^/df	CFI	TLI	RMSEA (90% CI)	SRMR	Model comparison	Δχ^2^ (Δdf)
M1: Four-factor model (hypothesized)	378.8	164	2.31	0.95	0.94	0.056 (0.048, 0.064)	0.045	—	—
M2: Three-factor model (OS + MD combined)	626.3	167	3.75	0.86	0.84	0.082 (0.074, 0.090)	0.078	M2 vs. M1	247.5 (3)***
M3: Three-factor model (EE + DC combined)	669.7	167	4.01	0.84	0.82	0.086 (0.078, 0.094)	0.082	M3 vs. M1	290.9 (3)***
M4: One-factor model	1011.2	170	5.95	0.72	0.69	0.110 (0.102, 0.118)	0.098	M4 vs. M1	632.4 (6)***

OS = occupational stress; MD = moral dilemmas; EE = emotional exhaustion; DC = decision-making confidence. ***p < 0.001.

Three alternative models were tested to compare discriminant validity: a one-factor model, a three-factor model combining occupational stress and moral dilemmas, and a three-factor model combining emotional exhaustion and decision-making confidence. All alternative models exhibited significantly worse fit than the hypothesized four-factor model (all Δχ^2^ *p* < 0.001), supporting the distinctiveness of the four constructs.

Standardized factor loadings, composite reliability (CR), and average variance extracted (AVE) are presented in Table 4. All standardized loadings were above 0.60 and statistically significant (*p* < 0.001). CR values ranged from 0.85 to 0.93, exceeding the 0.70 threshold for good reliability. AVE values ranged from 0.55 to 0.65, all above the 0.50 cutoff for acceptable convergent validity.

**Table 4:** Standardized factor loadings, composite reliability, and average variance extracted.

Construct	Indicator	Standardized loading	SE	t value	CR	AVE
Occupational stress (parceled)	Parcel 1 (items 1–5)	0.82	0.04	23.17***	0.88	0.65
	Parcel 2 (items 6–10)	0.79	0.04	21.42***		
	Parcel 3 (items 11–15)	0.76	0.05	18.95***		
	Parcel 4 (items 16–20)	0.85	0.04	24.68***		
Moral dilemmas	Item 1	0.72	0.05	15.32***	0.85	0.55
	Item 2	0.75	0.05	16.41***		
	Item 3	0.78	0.04	17.69***		
	Item 4	0.74	0.05	16.07***		
	Item 5	0.76	0.04	16.83***		
	Item 6	0.70	0.05	14.68***		
Emotional exhaustion	Item 1	0.78	0.04	19.21***	0.90	0.63
	Item 2	0.81	0.04	20.75***		
	Item 3	0.83	0.03	22.04***		
	Item 4	0.76	0.04	18.36***		
	Item 5	0.79	0.04	19.78***		
Decision-making confidence	Item 1	0.75	0.05	17.14***	0.88	0.60
	Item 2	0.79	0.04	18.87***		
	Item 3	0.82	0.04	20.12***		
	Item 4	0.73	0.05	16.29***		
	Item 5	0.77	0.04	18.05***		

SE = standard error; CR = composite reliability; AVE = average variance extracted. ***p < 0.001.

Discriminant validity was assessed using the Fornell-Larcker criterion. As shown in Table 5, the square root of the AVE for each construct was greater than the absolute value of its correlation with any other construct, confirming satisfactory discriminant validity.

**Table 5:** Discriminant validity test (Fornell-Larcker criterion).

Construct	1	2	3	4
1. Occupational stress	0.806			
2. Moral dilemmas	0.38	0.742		
3. Emotional exhaustion	0.49	0.41	0.794	
4. Decision-making confidence	−0.26	−0.24	−0.38	0.775

Diagonal values are the square roots of AVE; off-diagonal values are absolute correlation coefficients between constructs.

### 3.5 Structural model and mediation analyses

The structural model with duty type as a covariate exhibited good fit to the data: χ^2^/df = 2.26, CFI = 0.95, TLI = 0.94, RMSEA = 0.055 (90% CI [0.047, 0.063]), SRMR = 0.044.

Regarding the covariate, duty type significantly and positively predicted emotional exhaustion (β = 0.12, SE = 0.05, *p* = 0.018), indicating that front-line officers reported higher emotional exhaustion than non-front-line officers. Duty type did not significantly predict decision-making confidence (β = 0.08, SE = 0.05, *p* = 0.112).

Consistent with Hypothesis 1, occupational stress positively predicted emotional exhaustion (β = 0.41, SE = 0.05, *p* < 0.001). Supporting Hypothesis 2, moral dilemmas positively predicted emotional exhaustion over and above occupational stress (β = 0.27, SE = 0.05, *p* < 0.001). Emotional exhaustion in turn negatively predicted decision-making confidence (β = −0.38, SE = 0.06, *p* < 0.001). The model explained 38% of the variance in emotional exhaustion and 15% of the variance in decision-making confidence.

Bootstrap mediation tests with 5,000 bias-corrected resamples are summarized in Table 6. The indirect effect of occupational stress on decision-making confidence through emotional exhaustion was significant (indirect effect = −0.16, 95% CI [−0.21, −0.11]), while its direct effect was non-significant (direct effect = −0.02, 95% CI [−0.12, 0.08]), supporting Hypothesis 3a. Similarly, the indirect effect of moral dilemmas through emotional exhaustion was significant (indirect effect = −0.10, 95% CI [−0.15, −0.06]), with a non-significant direct effect (direct effect = −0.07, 95% CI [−0.16, 0.02]), supporting Hypothesis 3b. As a descriptive decomposition of the observed associations, the indirect effects corresponded to 88.9 and 58.8% of the total effects of occupational stress and moral dilemmas, respectively.

**Table 6:** Bootstrap tests of direct and indirect effects in the mediation model (5,000 resamples).

Path	Standardized effect	SE	95% CI	Conclusion
Occupational stress → Emotional exhaustion	0.41***	0.05	[0.31, 0.50]	H1 supported
Moral dilemmas → Emotional exhaustion	0.27***	0.05	[0.17, 0.37]	H2 supported
Emotional exhaustion → Decision-making confidence	−0.38***	0.06	[−0.49, −0.27]	Significant
Occupational stress → Decision-making confidence (direct)	−0.02	0.05	[−0.12, 0.08]	n.s.
Occupational stress → EE → Decision-making confidence (indirect)	−0.16	0.03	[−0.21, −0.11]	H3a supported
Moral dilemmas → Decision-making confidence (direct)	−0.07	0.05	[−0.16, 0.02]	n.s.
Moral dilemmas → EE → Decision-making confidence (indirect)	−0.10	0.02	[−0.15, −0.06]	H3b supported

EE = emotional exhaustion; SE = standard error; CI = confidence interval; n.s. = not significant. ***p < 0.001.

### 3.6 Robustness checks

Five robustness tests were conducted to assess the stability of the results:

Estimation method replacement. Re-estimating the model with listwise deletion instead of full information maximum likelihood produced path coefficients and indirect effects within 0.03 of the original values, with identical significance patterns.

Subsample analysis. Restricting the analysis to front-line officers only (*n* = 268) yielded the same pattern of significant indirect and non-significant direct effects. Occupational stress (β = 0.39, *p* < 0.001) and moral dilemmas (β = 0.29, *p* < 0.001) predicted emotional exhaustion, which predicted lower decision confidence (β = −0.36, *p* < 0.001). The indirect effects remained significant (OS: −0.14, 95% CI [−0.20, −0.09]; MD: −0.10, 95% CI [−0.16, −0.05]).

Additional covariate control. Adding psychological distress (GHQ-12) as a covariate did not alter the core findings. Psychological distress predicted both emotional exhaustion (β = 0.22, *p* < 0.001) and decision confidence (β = −0.11, *p* = 0.047), but all mediation paths remained significant and in the same direction, indicating that the mediation effect is independent of general psychological distress.

Bureau fixed effects. Re-estimating the structural model with dummy variables for the three participating bureaus (with Zhengzhou as the reference category) changed the standardized path estimates by at most 0.02 and left all significance conclusions unchanged, indicating that the results are not driven by between-bureau differences.

Sequential alternative model. As a sensitivity analysis, a sequential specification in which occupational stress was modeled as an antecedent of moral dilemmas, with moral dilemmas in turn predicting emotional exhaustion and decision-making confidence, fit the data worse than the hypothesized parallel model (χ^2^/df = 2.39 vs. 2.26; CFI = 0.94 vs. 0.95; RMSEA = 0.059 vs. 0.055), and the substantive conclusions were unchanged. Given the cross-sectional design, the data cannot adjudicate between these specifications; the parallel structure is retained on theoretical grounds (see Section 4).

## 4 Discussion

This study tested whether emotional exhaustion mediates the associations of occupational stress and moral dilemmas with decision-making confidence among Chinese police officers. The results supported all three hypotheses. Both occupational stress and moral dilemmas were positively associated with emotional exhaustion, and the direct effects of both antecedents on decision-making confidence were non-significant once emotional exhaustion was included, consistent with the hypothesized model. The findings offer three contributions to the psychological literature on policing and decision-making.

First, the study provides cross-sectional evidence from a non-Western police system for a resource-based account of the association between occupational strain and decision confidence. The positive association between occupational stress and emotional exhaustion (H1) is consistent with the JD-R framework and with meta-analytic evidence showing a robust stress-burnout link among Chinese officers (Demerouti et al., 2001; Zhou et al., 2024). The negative association between emotional exhaustion and decision-making confidence extends attentional control and resource theories from acute laboratory threat to chronic occupational depletion: just as experimentally induced anxiety biases shooting decisions (Nieuwenhuys et al., 2012a, 2012b, 2015), chronic depletion of energetic resources appears to erode officers’ self-assuredness in their judgments. From the perspective of COR theory, exhausted officers have fewer resources to invest in effortful, controlled decision processes, and their confidence in those processes declines accordingly (Hobfoll, 1989, 2001).

Second, the study quantifies moral dilemmas as an independent antecedent of burnout. That moral dilemmas predicted emotional exhaustion over and above a broad operational stress measure (H2) indicates that the moral dimension of policing is not reducible to workload or operational danger. This finding resonates with the moral injury literature, in which morally injurious experiences predict trauma symptomatology independently of conventional stressors (Papazoglou et al., 2020), and with qualitative evidence that Chinese officers actively manage moral ambivalence in enforcement contexts (Liu, 2023). It also extends the moral dilemma construct, previously examined largely through qualitative or vignette-based methods, to survey-based quantification in a large officer sample.

Third, the pattern of non-significant direct effects (H3) has practical significance. The absence of significant direct effects of occupational stress and moral dilemmas on decision confidence indicates that the negative associations of chronic strain with self-evaluated decisional capacity were statistically accounted for by emotional exhaustion. For police organizations, this suggests that burnout is relevant not only as a welfare or retention issue but also for officers’ self-evaluated decisional capacity: officers who are emotionally exhausted report less confidence in their judgments. It also identifies a single, measurable, and modifiable target, emotional exhaustion, on which diverse sources of strain converge. A recent meta-analysis found no pooled association between emotional exhaustion and officers’ use of force (Blomqvist Mickelsson et al., 2026), and whether the loss of self-evaluated decisional capacity documented here translates into objectively different decisions remains an open empirical question. Mental health support programs, stress management training, and workload redesign that reduce emotional exhaustion may therefore benefit officers’ self-evaluated decisional capacity even when the underlying stressors, such as shift work or morally demanding enforcement duties, cannot be eliminated. Prior intervention research has shown that burnout is responsive to organizational and individual-level interventions (Maslach et al., 2001); whether such interventions also affect decision-making behavior remains to be tested.

The findings also speak to the broader agenda of understanding the emotional and motivational processes that shape police judgment under pressure (Fridman et al., 2019). The present model demonstrates that two distinct classes of antecedents, one primarily organizational-operational (occupational stress) and one primarily moral-experiential (moral dilemmas), converge on a single emotional state. This convergence is theoretically important because it suggests that emotional exhaustion functions as a general resource-deficit state that transcends the specific content of the stressors that produce it, consistent with the resource-based logic of both COR theory and the JD-R model (Hobfoll, 1989; Demerouti et al., 2001). The non-significant direct paths are consistent with longitudinal evidence that job resources buffer the impact of demands on strain (Hu et al., 2017). At the same time, the stronger indirect effect of occupational stress, relative to moral dilemmas, is consistent with the primacy of workload in Chinese police settings, where chronic understaffing and administrative overload are among the most frequently reported stressors (Wu and Wen, 2020; Zhou et al., 2024).

Several implications for police training and support services follow directly from these results. First, because emotional exhaustion is the operative mechanism, burnout screening should be integrated into routine psychological health assessments for officers, using validated instruments such as the MBI-GS, rather than focusing exclusively on PTSD or depression symptomatology (Sherwood et al., 2019). Second, organizational interventions that reduce emotional exhaustion, including workload management, shift schedule design, supervisory support, and access to psychological services, should be evaluated not only for their effects on officer welfare but also for their downstream effects on self-reported decision-related outcomes. Third, the independent contribution of moral dilemmas suggests that officers would benefit from structured opportunities to process morally demanding experiences, for example, through ethics-focused debriefing or peer-support programs that normalize moral ambivalence and prevent the moral disengagement documented among Chinese tactical officers (Bandura, 1999; Liu, 2023; Papazoglou et al., 2020).

### 4.1 Limitations

Several limitations warrant consideration. First, the cross-sectional design precludes causal inference; although the hypothesized ordering is theoretically grounded, reciprocal relationships, for example, low decision confidence contributing to greater perceived stress, cannot be ruled out (Maxwell and Cole, 2007), and longitudinal or diary designs are needed to establish temporal precedence. Second, all measures were self-report. The Harman test reported in Section 3.2 is descriptive and does not rule out common method variance; marker-variable designs, supervisor ratings, simulated decision tasks, or physiological indicators of strain are needed in future work (Podsakoff et al., 2003). Third, the moral dilemma and decision-making confidence scales were developed for this study; although their psychometric properties were adequate and their content was grounded in established literatures, further validation in independent samples is required. Fourth, the convenience sample, drawn from three municipal bureaus in one province, limits generalizability, although the demographic profile broadly resembled that of recent Chinese police samples (Wu and Wen, 2020). Finally, the decision-making confidence construct captures officers’ self-assessed confidence rather than objective decision accuracy; establishing the link between exhaustion-related loss of confidence and actual decision performance remains an important agenda for simulation-based research (Nieuwenhuys et al., 2015; Fridman et al., 2019).

Notwithstanding these limitations, the present study indicates that, within the hypothesized model, the associations of occupational stress and moral dilemmas with decision-making confidence among Chinese police officers operated through emotional exhaustion: once exhaustion was accounted for, neither antecedent showed a significant direct effect. The findings bridge the occupational health and police decision-making literatures, extend resource-based theories of strain to the domain of judgment, and identify emotional exhaustion as a candidate target for interventions aimed at officers’ self-evaluated decisional capacity. Future research should test the model longitudinally, incorporate objective and physiological indicators of strain and decision performance, and evaluate whether burnout-focused interventions preserve decision confidence in operational settings.

## Data Availability

The original contributions presented in the study are included in the article/Supplementary material, further inquiries can be directed to the corresponding author.
