# Supplementary material for: Occupational stress, moral dilemmas, and decision-making confidence among Chinese police officers: the mediating role of emotional exhaustion
Source: Front Psychol. 2026 Sep 30;17:1971012. doi: 10.3389/fpsyg.2026.1971012 (PMC13661478; doi:10.3389/fpsyg.2026.1971012)
Supplement: Supplementary file 1 [file Data_Sheet_1.PDF]

Supplementary Material

for

Occupational Stress, Moral Dilemmas, and Decision-Making Confidence Among Chinese Police Officers: The Mediating Role of Emotional Exhaustion

Yi Li
Mental Health Education Center, Henan Police College, Zhengzhou, China

This document reports the full item wording, in English and in the administered Chinese, of the two measures developed for the present study: the six-item Moral Dilemmas Scale and the five-item Decision-Making Confidence Scale. All other instruments (Operational Police Stress Questionnaire, emotional exhaustion subscale of the Maslach Burnout Inventory-General Survey, and the 12-item General Health Questionnaire) are published measures cited in the main text.

S1. Moral Dilemmas Scale (six items)

Construct definition. The scale measures the frequency with which officers encounter situations in which duties, values, or loyalties conflict, such that acting in accordance with one duty necessarily violates another. The construct is distinct from moral distress and moral injury, which refer to the psychological aftermath of such events rather than to the exposure itself.

Response format. Each item is rated on a five-point frequency scale referring to the past six months: 1 = never (从未), 2 = rarely (很少), 3 = sometimes (有时), 4 = often (经常), 5 = very often (非常频繁). The scale score is the mean of the six items, with higher scores indicating more frequent exposure to moral dilemmas.

Item 1. In the past six months, how often did you face situations in which following orders conflicted with what you personally believed was right?
（条目 1 中文施测版）过去六个月，您是否面临过执行命令与个人信念相冲突的情况？

Item 2. In the past six months, how often did you encounter situations in which strict enforcement of regulations could harm the interests of a vulnerable citizen?
（条目 2 中文施测版）过去六个月，您是否遇到过严格执行规定可能损害弱势群体利益的情形？

Item 3. In the past six months, how often did you have to balance enforcement targets against the interests of minor offenders?
（条目 3 中文施测版）过去六个月，您是否不得不在执法目标与轻微违法者利益之间做出权衡？

Item 4. In the past six months, how often did you witness or participate in actions that conflicted with your personal moral standards?
（条目 4 中文施测版）过去六个月，您是否目睹或参与过与个人道德标准相冲突的行动？

Item 5. In the past six months, how often were you required by your job to do something you considered unjust?
（条目 5 中文施测版）过去六个月，您是否因工作要求而不得不做内心认为不公正的事？

Item 6. In the past six months, how often did you have to choose between conflicting duties or loyalties?
（条目 6 中文施测版）过去六个月，您是否经常需要在相互冲突的职责或忠诚之间做出选择？

Psychometric properties in the present sample: unidimensional structure (CFI = .97, RMSEA = .06); Cronbach's α = .84.

S2. Decision-Making Confidence Scale (five items)

Construct definition. The scale measures officers' self-reported evaluation of their own decisional capacity. The construct is distinct from decision accuracy, confidence calibration, decisiveness, and general self-efficacy.

Response format. Each item is rated on a five-point agreement scale: 1 = strongly disagree (非常不同意), 2 = disagree (不同意), 3 = neutral (不确定), 4 = agree (同意), 5 = strongly agree (非常同意). The scale score is the mean of the five items, with higher scores indicating greater self-evaluated decisional capacity.

Item 1. Even in uncertain situations, I am confident in my ability to make the right decision.
（条目 1 中文施测版）即使在不确定的情况下，我也能做出正确的决定。

Item 2. Under time pressure, I am able to remain decisive.
（条目 2 中文施测版）面对时间压力，我能保持果断。

Item 3. I am able to handle situations proportionately in accordance with law and policy.
（条目 3 中文施测版）我能根据法律和政策比例适当地处理情况。

Item 4. After making a decision, I rarely doubt whether it was correct.
（条目 4 中文施测版）做出决定后，我很少怀疑自己是否正确。

Item 5. I trust my own judgment in my work.
（条目 5 中文施测版）我信任自己在工作中的判断能力。

Psychometric properties in the present sample: unidimensional structure (CFI = .98, RMSEA = .05); Cronbach's α = .86.
